# Illumination of serotonin transporter mechanism and role of the allosteric site

**DOI:** 10.1126/sciadv.abl3857

**Published:** 2021-12-01

**Authors:** Dongxue Yang, Eric Gouaux

**Affiliations:** 1 Vollum Institute, Oregon Health and Science University, Portland, OR 97239, USA.; 2 Howard Hughes Medical Institute, Oregon Health and Science University, Portland, OR 97239, USA.

## Abstract

The serotonin transporter (SERT) terminates serotonin signaling by using sodium and chloride gradients to drive reuptake of serotonin into presynaptic neurons and is the target of widely used medications to treat neuropsychiatric disorders. Despite decades of study, the molecular mechanism of serotonin transport, the coupling to ion gradients, and the role of the allosteric site have remained elusive. Here, we present cryo–electron microscopy structures of SERT in serotonin-bound and serotonin-free states, in the presence of sodium or potassium, resolving all fundamental states of the transport cycle. From the SERT-serotonin complex, we localize the substrate-bound allosteric site, formed by an aromatic pocket positioned in the scaffold domain in the extracellular vestibule, connected to the central site via a short tunnel. Together with elucidation of multiple apo state conformations, we provide previously unseen structural understanding of allosteric modulation, demonstrating how SERT binds serotonin from synaptic volumes and promotes unbinding into the presynaptic neurons.

## Teaser

Cryo-EM and binding experiments reveal the molecular mechanism of serotonin transport by the human serotonin transporter.

## INTRODUCTION

Serotonin [5-hydroxytryptamine (5-HT)] is a ubiquitous chemical neurotransmitter in both the central and peripheral nervous systems. In the central nervous system, 5-HT exerts a profound effect on pain, mood, sleep, appetite, and attention, whereas 5-HT activity in the peripheral nervous system modulates peristalsis, mucus production, and blood vessel dilation (*1*). Although there are a diverse arrays of receptors that mediate the functional effects of 5-HT (*2*), its activity is extinguished by just one molecular species, the Na^+^/Cl^−^–coupled serotonin transporter (SERT) (*3*). In accord with the crucial role that 5-HT plays in mood states, selective serotonin reuptake inhibitors that act on SERT are some of the most widely used medications to treat depression, obsessive compulsive disorder, fibromyalgia, and anxiety disorders (*4*). SERT is also the target of a number of different abused and illicit psychostimulants, including cocaine and amphetamines (*5*).

SERT belongs to the neurotransmitter sodium symporter (NSS) family that includes the dopamine (DAT), norepinephrine (NET), glycine (GlyT1 and GlyT2), and γ-aminobutyric acid (GABA) transporters. The amino acid sequences and three-dimensional (3D) structures of NSS family members are closely related to the prokaryotic amino acid transporters LeuT and MhsT (*3*). Extensive structural and functional studies on LeuT (*6*) and MhsT (*7*), together with recent investigations of SERT (*8*–*11*) and GlyT1 (*12*), have shown how an inverted pseudo-repeat of five transmembrane (TM) helices creates a substrate-binding site approximately halfway across the membrane together with adjacent ion-binding sites. These binding sites become alternately accessible to the extracellular and intracellular spaces as transporters transition between different states in their transport cycles.

SERT (*8*) and DAT (*13*) have a second binding site for small molecules in an extracellular-exposed region of the transporter. In SERT, occupancy of this site by antidepressants such as imipramine (*14*) and citalopram (*8*), or by serotonin (*15*), slows the unbinding of ligands from the central site, suggesting that the second extracellular site is allosteric in nature. In DAT, and recently in SERT, small molecules targeting the allosteric site are validated reuptake inhibitors (*13*) and, in the case of SERT, clinically prescribed antidepressants (*16*, *17*). While the role of the allosteric site in SERT and DAT in slowing the unbinding of ligands from the central site and in inhibiting transport is unequivocal, the postulated presence of an allosteric site in the prokaryotic NSS homologs LeuT and MhsT, and the possible role of the allosteric site in these bacterial transporters, is controversial (*18*–*22*). The proposed role of substrate binding to the allosteric site in LeuT (*18*) and MhsT (*22*) as “triggering” release of the substrate from the central site to the cytoplasm has been extended to mechanistic schemes for eukaryotic NSSs, including SERT, without any direct evidence for the role of substrate binding to the allosteric site. Thus, the extent to which the substrate occupies the allosteric site in SERT throughout the transport cycle and whether occupancy of the allosteric site by the substrate affects substrate transport are presently unknown.

Despite intense interest, however, there is no structure-based understanding of the mechanism by which the substrate is transported from the extracellular to the intracellular space for any NSS family member, largely due to challenges posed by the small size and labile nature of these transporters. In particular, the location of the second substrate-binding site, how it is occupied by the substrate, and how the two substrate-binding sites participate in substrate transport are without structural knowledge. Moreover, the crucial roles of Na^+^ and Cl^−^ in modulating the conformational changes that drive substrate reuptake and the participation of K^+^ in returning SERT to an outward-facing conformation also have no foundation in 3D structure. Here, we develop methods to reconstitute human SERT, active in substrate and ligand-binding activity, into membrane bilayer-like nanodiscs filled with brain lipid. Using the high-affinity 15B8 Fab, we devise a framework to carry out high-resolution, single-particle cryo–electron microscopy (cryo-EM) reconstructions and complementary binding assays. Our results provide structural insight into the molecular mechanism of ion-coupled serotonin reuptake by SERT.

## RESULTS AND DISCUSSION

### 5-HT binds to two sites in the presence of Na^+^

**Fig. 1.**
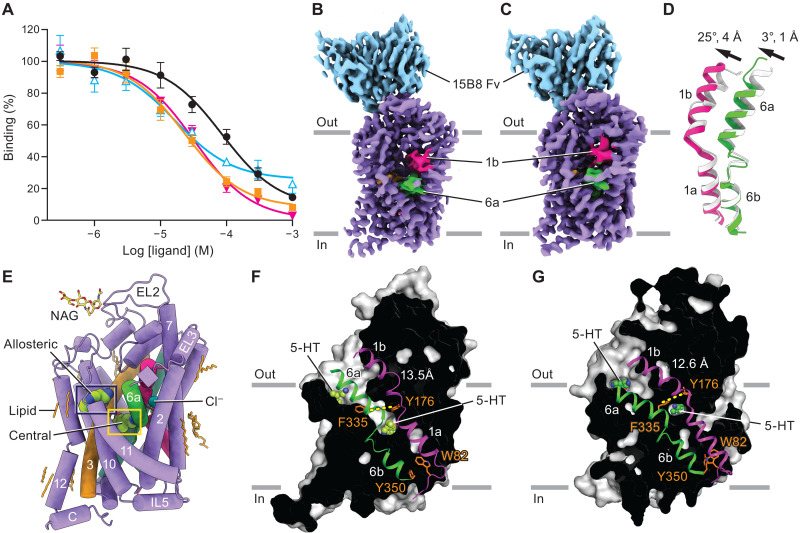
Mechanism of 5-HT capture. (**A**) Competition binding of 5-HT (black circles), *N*-ω-methyl-5-hydroxytryptamine (blue triangles), 5-methoxy-*N*-methyltryptamine (orange squares), and 3-(2-aminoethyl)-1-methyl-1*H*-indol-5-ol hydrochloride (magenta triangles) with [^3^H]paroxetine for SERT reconstituted into nanodiscs in the presence of 100 mM NaCl yields *K*_i_ values of 14.2 ± 3.0 μM, 2.8 ± 0.9 μM, 3.9 ± 0.9 μM, and 5.4 ± 1.0 μM, respectively. Symbols show the mean values derived from *n* = 3 technical replicates. Error bars show the SEM. (**B** and **C**) Cryo-EM maps of 5-HT in complex with SERT, bound with 15B8 Fab in NaCl in the outward-facing (B) and occluded (C) conformations. The constant domain of 15B8 Fab was masked out. (**D**) Displacements of TM6a and TM1b from the 5-HT bound outward-facing (gray) to the occluded conformations. Distance measurements were made using Cα positions of Y186 in TM3 to Q111 in TM1 and D328 in TM6. The TM1 to TM12 helices were superimposed, and the angle between the helices was measured using Cα positions in PyMOL. (**E**) Overall structure of the 5-HT complex in NaCl in the outward-facing conformation, shown in cartoon representation. (**F** and **G**) “Slab” views of the extracellular and intracellular cavities of the 5-HT–SERT complex in the outward (F) and occluded (G) conformations. Distances between the residues (F335 and Y176) defining the extracellular gate are indicated.

We first validated 5-HT binding to SERT in nanodiscs via [^3^H]paroxetine competition–binding experiments in the presence of NaCl, which yielded an inhibitory constant (*K*_i_) of 14 ± 3 μM (Fig. 1A). We then carried out a single-particle cryo-EM reconstruction of the 5-HT–bound SERT complex in the same conditions (fig. S1). After careful 3D classification, we found two classes of SERT that differed in the vicinity of the extracellular gate (Fig. 1, B and C; fig. S2; and Table 1). The two classes adopt outward-open and occluded conformations based on the “tilting” of TM1b and TM6a relative to the scaffold domain, conformational differences that open or occlude the central binding site, respectively (Fig. 1D). With all TM helices and most side chains well resolved, TM1a of both classes adopts a closed conformation, occluding the permeation pathway to the intracellular side (Fig. 1, E to G). The outward-open class in which the extracellular gate is open (87% of particles) resembles the outward-open state of the (*S*)-citalopram–bound complex [Protein Data Bank (PDB) code: 5I73; Fig. 1F and fig. S3A] (*8*). The occluded class in which both the extracellular and intracellular gates are closed (13% of particles) and comparison of the positions of TM1, TM6, and the extracellular gate to the equivalent elements of the ibogaine-bound occluded complex (*10*) (PDB code: 6DZV) indicate that TM1b and TM6a shift toward the scaffold domain, thus yielding an occluded conformation (Fig. 1G and fig. S3B).

**Table 1.** Cryo-EM data collection, refinement, and validation statistics.

	**5-HT–SERT** **outward-NaCl** **(EMD-23365)** **(PDB 7LIA )**	**5-HT–SERT** **occluded-NaCl** **(EMD-23830)** **(PDB 7MGW)**	**5-HT–SERT** **inward-KCl** **(EMD-23364)** **(PDB 7LI9)**	**Apo SERT** **occluded-NaCl** **(EMD-23362)** **(PDB 7LI7)**	**Apo SERT** **inward-NaCl** **(EMD-23363)** **(PDB 7LI8)**	**Apo SERT** **inward-KCl** **(EMD-23361)** **(PDB 7LI6)**
Data collection and processing						
Magnification	77,160		62,383	77,160		75,642
Voltage (kV)	300		300	300		300
Electron exposure (e−/Å^2^)	55		57	50		52
Defocus range (μm)	−0.8 to −2.5		−0.9 to −2.7	−1.0 to −2.5		−1.0 to −2.5
Pixel size (Å)	0.648		0.802	0.648		0.661
Symmetry imposed	C1	C1	C1	C1	C1	C1
Initial particle images (no.)	2,861,741		5,624,930	5,568,431		5,865,511
Final particle images (no.)	774,907	114,222	299,808	636,335	343,085	707,210
Map resolution (Å)	3.1	3.5	3.7	3.9	3.9	3.5
FSC threshold	0.143	0.143	0.143	0.143	0.143	0.143
Map resolution range (Å)	3.4–2.6	3.8–3.0	4.0–3.0	4.0–3.2	4.2–3.4	3.6–3.0
Refinement						
Initial model used (PDB code)	6DZW	6DZV	6DZZ	6DZV	6DZZ	6DZZ
Initial model CC	0.59	0.78	0.83	0.56	0.74	0.64
Model resolution (Å)	3.5	4.0	3.9	4.1	4.2	3.8
FSC threshold	0.5	0.5	0.5	0.5	0.5	0.5
Map sharpening *B* factor (Å^2^)	−178	−157	−161	−214	−195	−185
Model composition						
Nonhydrogen atoms	6,279	6,223	6,174	6,177	6,181	6,249
Protein residues	767	765	767	765	767	767
Ligands (atoms)	231	188	128	142	137	224
*B* factors (Å^2^)						
Protein	28	192	29	98	99	73
Ligand	28	180	22	89	91	76
Root mean square deviations						
Bond lengths (Å)	0.002	0.002	0.002	0.002	0.002	0.002
Bond angles (°)	0.422	0.470	0.444	0.470	0.461	0.444
Validation						
Refined model CC	0.72	0.84	0.71	0.67	0.79	0.71
MolProbity score	1.71	1.75	1.70	1.81	1.74	1.81
Clashscore	7.98	8.97	6.00	10.00	6.76	5.29
Poor rotamers (%)	1.71	1.40	0.00	0.00	0.00	1.72
Ramachandran plot						
Favored (%)	97.50	97.10	94.61	95.78	94.74	95.01
Allowed (%)	2.50	2.90	5.39	4.22	5.26	4.99
Disallowed (%)	0	0	0	0	0	0

**Fig. 2.**
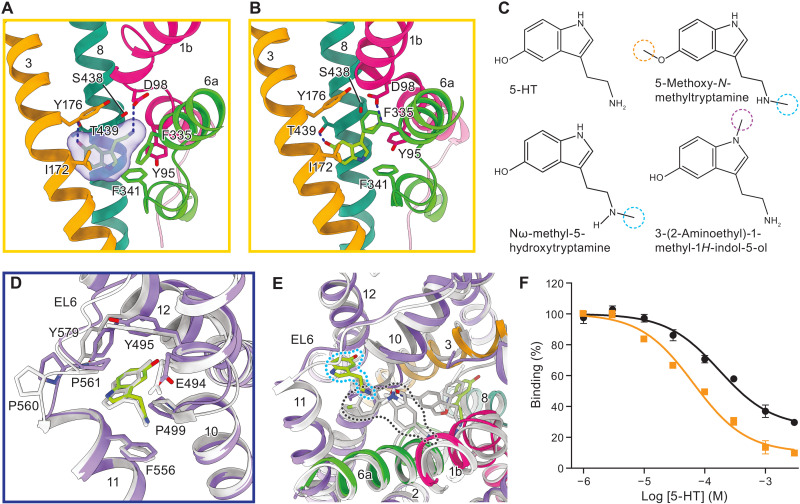
5-HT binds to central and allosteric sites. (**A** and **B**) 5-HT interactions within the central binding site for the outward (A) and occluded (B) conformations. Hydrogen bonds formed between 5-HT and D98 and T439 are indicated with dark blue dashed lines. (**C**) Chemical structures of 5-HT and methyl analogs, with the methyl groups circled. (**D**) Comparison of 5-HT binding pose and interactions with surrounding residues in the allosteric site in the outward (purple) and occluded (gray) conformations. (**E**) Comparison of 5-HT binding in the allosteric site with *S*-citalopram binding in the extracellular vestibule (PDB code: 5I73, gray). (**F**) Plots of competition binding of 5-HT (black circles) and the *N*-indole methyl derivative (orange squares) against [^3^H]VLZ in NaCl. Data are means ± SEM.

Our well-resolved density map permitted the identification of densities for both Na^+^ and Cl^−^ in the outward-facing conformation, confirming the positions and coordinating residues that were previously observed in an x-ray structure of SERT in complex with the antidepressant (*S*)-citalopram (fig. S3, C to F) (*8*). The density map also enabled assignment of a density in the central binding site as 5-HT in both outward and occluded conformations (Fig. 2, A and B, and fig. S2, H and L). Further inspection revealed that 5-HT forms similar interactions with SERT in both conformations: The tryptamine group of 5-HT is lodged between the aromatic groups of Tyr^176^ and Tyr^95^, and the primary amine interacts with Ser^438^ and forms a hydrogen bond with Asp^98^, a residue highly conserved among biogenic amine neurotransmitter transporters (*23*), as predicated previously (*24*) and as seen with the hydrogen bond formed between the amine group of dopamine and the equivalent Asp^46^ residue in DAT (PDB code: 4XP1) (*25*). In SERT, the hydroxyl group inserts into the space between TM3 and TM8 and is coordinated by the side chains of Thr^439^, Ala^173^, and Ala^169^, whereas in DAT, the catechol ring of dopamine interacts with TM3 and TM8 by hydrogen bonds with the carboxylate group of Asp^121^ and carbonyl oxygen of Ala^117^ (*25*). The indole ring forms a nonpolar interaction with the crucial Ile^172^ residue that lies “above” it (*26*), and the indole nitrogen forms an aromatic interaction with Phe^341^. In contrast, we noted that Phe^335^ undergoes a conformational change between the outward and occluded conformations that causes it to move further into the central binding site (Fig. 2, A and B, and fig. S3F).

Because SERT transports other biogenic amines, including dopamine (*27*), we investigated the size and shape of the central site in the 5-HT complex. Notably, 5-HT has a “loose” fit to the central site, neither spanning the entire binding pocket nor fully occupying the volume of the central site (Fig. 2A). We therefore speculated that SERT should tolerate 5-HT analogs augmented with methyl groups (Fig. 2C) and tested this hypothesis using a [^3^H]paroxetine competition–binding assay. All three single methyl 5-HT analogs that we tested competed for paroxetine binding and had a two- to fourfold higher binding affinity (Fig. 1A). We further demonstrated that the ^3^H-labeled *N*-methyl indole derivative is a robust substrate for SERT (fig. S3H). These experiments support the pose that 5-HT adopts in our structures and the spacious nature of the central site, which likely underlies SERT’s promiscuous substrate specificity.

Inspired to find the location of the long-sought 5-HT allosteric site, we scrutinized the density maps of the 5-HT–bound SERT complex and found a nonprotein density in a hydrophobic pocket within the scaffold domain in both outward-facing and occluded conformations. This density had a similar shape to that in the central site and was well fitted by a second 5-HT molecule (fig. S2, H and L). The binding site is primarily formed by TM10 to TM12 and extracellular loop 6 (EL6), and its size and shape are similar in both outward and occluded conformations (Fig. 2D). Key features include protrusion of the 5-HT hydroxyl group into the space between TM10 and TM12, a Pro^560^-Pro^561^ motif in EL6 that delineates the “upper” portion of the site, and cradling of the ligand by Pro^499^. Tyr^495^ from TM10 and Tyr^579^ from TM12 also engage in aromatic interactions with the indole ring, and Glu^494^ in TM10 forms a hydrogen bond with the primary amine group of 5-HT. The side chain of Phe^556^ does not occupy the binding site as it does in the corresponding apo structures. Instead, it is apparently displaced by the 5-HT molecule and interacts with the amine group of 5-HT from underneath (Fig. 2D). The initial studies of the allosteric site in SERT showed how a range of chemically and structurally diverse small molecules slow the unbinding of radiolabeled ligands from the primary or central site (*28*), thus foreshadowing the plasticity of the site. In accordance with the promiscuity suggested by these studies, we found that 5-HT binds to the same overall region as (*S*)-citalopram, but it is lodged more deeply into the “cradle” defined by TM10 to TM12 and does not directly occlude egress of 5-HT from the central site (Fig. 2E).

To validate binding of 5-HT at the allosteric 5-HT site, we performed competition-binding experiments using [^3^H]vilazodone ([^3^H]VLZ), a recently developed and widely prescribed antidepressant predicted to bind to the allosteric site (*17*). We initially carried out saturation binding experiments in the presence of imipramine, which binds to the central site at nanomolar concentrations (*16*), and recorded a dissociation constant (*K*_d_) of 306 ± 36 nM for [^3^H]VLZ (fig. S3I). Subsequently, competition-binding experiments in the presence of both [^3^H]VLZ and imipramine, using increasing concentrations of 5-HT, resulted in a *K*_i_ measurement of 48 ± 12 μM, which represents an estimate of 5-HT’s affinity for the allosteric site (Fig. 2F). Furthermore, having noted that the indole nitrogen of 5-HT is surrounded by hydrophobic residues in the allosteric site (Fig. 2D), we reasoned that the addition of a methyl group to the indole nitrogen would enhance binding to this site. As predicted, 3-(2-aminoethyl)-1-methyl-1*H*-indol-5-ol hydrochloride exhibited a higher binding affinity (*K*_i_ = 24.9 ± 5.4 μM; *P* < 0.05, one-sided Student’s *t* test) than 5-HT (Fig. 2F), consistent with the assigned binding pose of 5-HT.

Together, our data reveal that in the presence of Na^+^, 5-HT binds to two distinct sites on SERT: a central, orthosteric binding site and a peripheral, allosteric binding site. However, unlike small-molecule antidepressants, 5-HT binds within the scaffold domain at a position that does not directly occlude unbinding of ligands from the central site. We speculate that the mechanism by which 5-HT binding to allosteric site slows ligand unbinding from the central site is not via direct steric interference but rather via indirect actions, which might include stabilization of occluded conformations.

### An inward-facing conformation in a Na^+^-lacking, 5-HT/K^+^–containing buffer

**Fig. 3.**
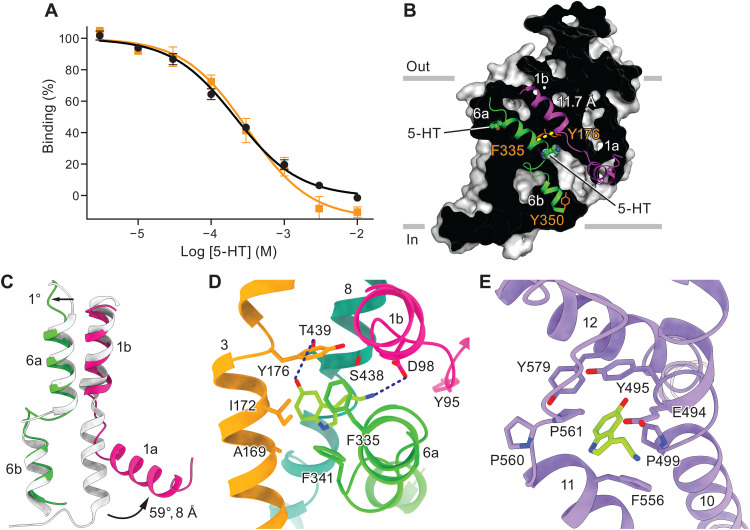
Molecular insights into 5-HT release. (**A**) Competition binding of 5-HT against [^3^H]ibogaine in the presence of NaCl (black, circles) or KCl (orange, squares). Error bars show the SEM. (**B**) Slab view of the extracellular and intracellular cavities of the 5-HT–SERT complex in KCl. (**C**) Comparison of TM1 and TM6 in the 5-HT bound inward and occluded (gray) conformations. Distance measurements were made using Cα positions of G160 in TM3 to W85 in TM1a. (**D** and **E**) 5-HT recognitions in the central site (D) and allosteric site (E).

K^+^ is counter-transported by SERT (*3*, *29*), and studies of LeuT have demonstrated that K^+^ influences conformational transitions by inducing closure of the extracellular gate (*30*). K^+^ has also been found to substantially increase the accessibility of TM5 due to the intracellular gate opening in a concentration-dependent manner (*31*). We therefore carried out structural and binding studies in the presence of 100 mM KCl and in the absence of Na^+^ to investigate how 5-HT is released into the cytoplasm and to illuminate the role of the allosteric site during the transport cycle. Exploiting the fact that ibogaine binding is Na^+^ independent (*10*), we conducted [^3^H]ibogaine competition–binding experiments in KCl and measured a similar *K*_i_ value for 5-HT (198 ± 36 μM) to that measured in NaCl (171 ± 22 μM; Fig. 3A), underscoring the conclusion that 5-HT binding is Na^+^ independent (*32*). We subsequently determined the structure of the 5-HT complex in these substrate-release conditions. The resulting single-particle reconstruction yielded a single 3D class, in which SERT adopted an inward-facing conformation, that we refined to ~3.7-Å resolution (Fig. 3B; figs. S4 and S5, A and B; and Table 1). Compared to the occluded conformation of 5-HT–bound SERT in NaCl, the extracellular pathway in KCl is closed by the movement of TM6a toward the scaffold domain (Fig. 3C). TM1a splays “outward” from the intracellular region of SERT, enabling displacement of Tyr^95^ and thus removing the barrier for 5-HT release into the cytoplasm (Fig. 3, C and D).

There is a strong density for 5-HT in the central site within this inward-facing conformation (Fig. 3D and fig. S4G). Moreover, although the extracellular vestibule is collapsed because of closure of the extracellular gate, there is also a strong density for 5-HT in the allosteric 5-HT site (fig. S4G). Thus, upon transition of the transporter from its outward-facing to occluded and then inward-facing conformation, the structural integrity of the allosteric site remains intact and 5-HT remains bound (Fig. 3E). The presence of 5-HT in the allosteric 5-HT site does not appear to affect extracellular gate closure or conformational transition of the transporter, although more precise measurements of transporter equilibria are required to confirm this inference. We suggest that 5-HT remains bound to the allosteric 5-HT site after extracellular gate closure because the 5-HT–bound site is sufficiently small to be accommodated within the scaffold domain. Single-molecule studies on MhsT (*22*) are consistent with a second substrate binding to the allosteric site and remaining trapped until the transporter cycles back to an outward-facing conformation.

Further examination of the two 5-HT–binding sites in the outward-facing, occluded, and inward-facing conformations revealed a tunnel between the allosteric 5-HT site and the central site in the outward-facing conformation (fig. S5, C and D). Thus, the substrate may access the central site from both the extracellular solution and the allosteric site, addressing an outstanding question about the role of the two binding sites in substrate transport. Previous studies of MhsT, which transports hydrophobic L-amino acids (*33*), have shown that it displays distinct transport rates for different substrates (*22*), implying that occupation of the allosteric site modulates substrate transport. However, we observe that the allosteric 5-HT site remains occupied and structurally stable in outward, occluded, and inward conformations. We hypothesize that the substrate could diffuse from the allosteric site to the central site when the transporter returns to the outward-facing conformation, efficiently triggering the next transport cycle.

### Apo-SERT is inward-facing in KCl in the absence of Na^+^

**Fig. 4.**
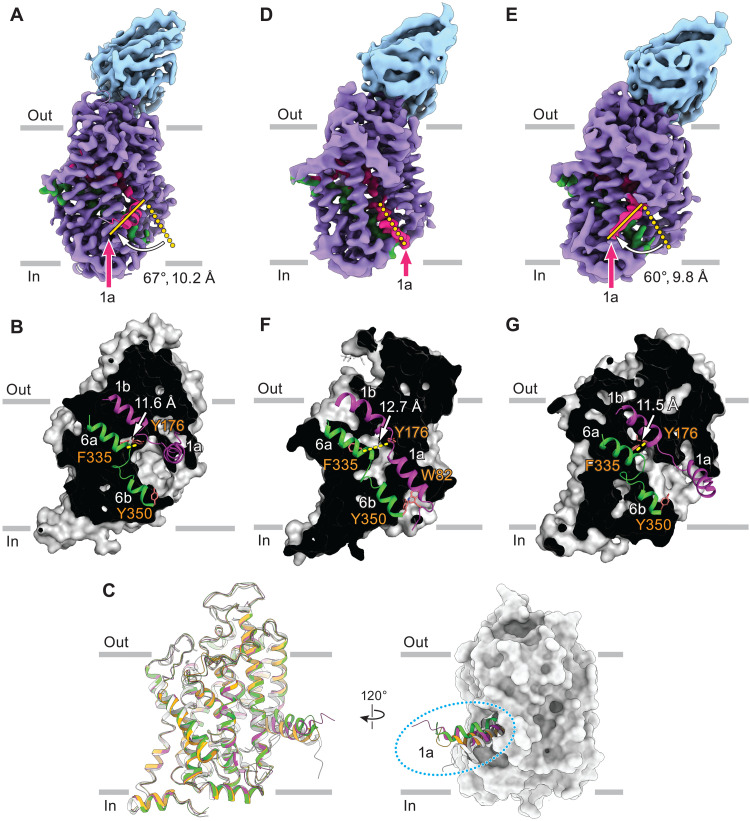
Cryo-EM reconstructions of apo SERT in KCl and NaCl. (**A**) The 3D reconstruction density map of apo SERT in KCl, bound to 15B8 Fab, adopted an inward-facing conformation. (**B**) Slab views of the extracellular and intracellular cavities of apo SERT in KCl. (**C**) Superposition of the inward-facing conformations of apo SERT in NaCl (green), apo SERT in KCl (magenta), 5-HT–bound SERT in KCl (yellow), and ibogaine-bound SERT in KCl (PDB code: 6DZZ, gray). (**D** and **E**) Cryo-EM maps of apo SERT in NaCl, bound with 15B8 Fab, captured in occluded (D) and inward (E) conformations. (**F** and **G**) Slab views of the extracellular and intracellular cavities of the occluded (F) and inward (G) conformations from apo SERT in NaCl.

We next determined the structure of SERT in the presence of K^+^ but in the absence of 5-HT and Na^+^, conditions under which SERT releases 5-HT into the cytoplasm and becomes primed to return to an outward-facing conformation. We overcame the difficulties associated with isolating apo SERT by purifying the transporter in the presence of 5-HT, reconstituting it into nanodiscs, and then removing weakly bound 5-HT by size exclusion chromatography (SEC) purification. Robust [^3^H]ibogaine binding verified that this SERT preparation is both active and apo (fig. S5E). The resulting cryo-EM reconstruction of apo SERT bound to 15B8 Fab in KCl revealed a single class with an inward-open conformation at 3.5-Å resolution, in which the extracellular gate is closed and the central binding pocket is solely accessible to the intracellular solution (Fig. 4, A and B; fig. S6; and Table 1). TM1a adopts a slightly different conformation to the 5-HT–bound inward-open state in KCl and is more open than the inward-open SERT-ibogaine complex (Fig. 4C) (*10*). The observation that TM1a samples many different orientations after opening of the intracellular gate is consistent with the heterogeneity found for TM1a in molecular dynamic (MD) simulations of LeuT (*34*) and underscores the highly dynamic nature and flexibility of this crucial structural element.

Together, our results suggest that SERT favors an inward-facing conformation with concomitant closure of the extracellular gate in the absence of Na^+^ and in the presence of K^+^. Unfortunately, we could not find unambiguous density for K^+^, leaving the important issues of where K^+^ binds, how it may stabilize the inward-open state, or whether it is simply the absence of Na^+^ that favors the inward-facing conformation unresolved.

### Na^+^ confers a dynamical behavior upon apo SERT

To understand how Na^+^ might prime SERT for subsequent 5-HT capture, we investigated the structural conformations of apo SERT in the presence of NaCl. We collected two independent cryo-EM datasets of the apo SERT–15B8 Fab complex and, after extensive 3D classification, found two classes with different positioning of TM1a (Fig. 4, D and E, and fig. S7), hereafter denoted as class 1 and class 2. Both reconstructions reached a resolution of ~3.9 Å (fig. S7 and Table 1) and exhibited distinct conformations of TM1 and TM6 compared to outward-open structures (*8*, *10*). In class 1 (~55%), TM1a adopts a closed intracellular gate conformation that prevents exposure of the central site to the cytoplasm (Fig. 4, D and F). In class 2 (~30%), TM1a is splayed out toward the membrane, opening the intracellular pathway to the cytoplasm (Fig. 4, E and G). Closure of the extracellular gate in both reconstructions prevents accessibility of the central site to the extracellular space (Fig. 4, F and G). The structure of class 1 is nearly identical to the occluded SERT-5-HT complex, with a root mean square deviation value of 0.4 Å for all TM Cα atoms. Accordingly, we define this class as having an occluded conformation. Because of the hinge-like movement of TM1a relative to the occluded state, which opens the intracellular gate, we define class 2 as having an inward-open conformation (Fig. 4, E and G). TM1a is tilted further “up” toward TM2 in this class compared to the inward-open SERT-ibogaine structure (*10*), allowing solvent access to the central site from the intracellular space (Fig. 4C).

The presence of multiple well-resolved classes of apo SERT in the presence of Na^+^ suggests that, in the absence of the substrate, the transporter adopts a range of conformations, including outward-open states. We propose that Na^+^-bound apo SERT is in a dynamic equilibrium with multiple conformations and that, once the transporter visits an outward-facing conformation and 5-HT binds to the central site from either bulk solution or the allosteric site, the transporter becomes primed for a cycle of substrate translocation.

### Mechanism of 5-HT transport

The allosteric site in SERT (*8*, *35*, *36*) was characterized via the observation that binding of divergent small molecules to this site could slow the dissociation of the ligand from the central site. An allosteric site for inhibitors has been defined and experimentally validated for other neurotransmitter transporters, including DAT (*37*) and NET (*28*), yet for the prokaryotic NSS homologues MhsT (*22*) and LeuT (*18*, *38*), the existence and role of an allosteric site are without structural validation and remain controversial. Nevertheless, the existence and location of the allosteric binding site, and our understanding of allosteric regulation in NSS transporters, is incomplete (*8*, *18*–*22*). Before the studies reported here, extensive structural studies of NSS family members have only revealed inhibitors or detergent molecules bound within the extracellular vestibule (*8*, *17*, *38*). Here, we provide structural insight into how 5-HT occupies an allosteric site in SERT, demonstrating that this second substrate site is near, but not completely overlapping with, the previously described allosteric antidepressant binding site (*8*, *35*, *36*). The structure of this site accommodates 5-HT within the aromatic pocket without hindering extracellular gate closure during the conformational transition. 5-HT remains bound to the allosteric 5-HT site during transport, and we speculate that it could diffuse to the central site once the transporter switches to the outward-facing conformation to rapidly trigger substrate transport. Given their structural and functional similarities, it is possible that all eukaryotic NSS family members have a similar allosteric site. The intact nature of the allosteric 5-HT site, in outward-facing, occluded, and inward-facing conformations, suggests that 5-HT bound at the allosteric site does not couple to 5-HT transport, distinct from the proposed nature and role of the putative allosteric site in MhsT (*22*) and LeuT (*18*). The second substrate-binding site in prokaryotic NSS homologues has remained contentious, and the proposed allosteric site is directly above the central site (*18*), which becomes “collapsed” upon closure of the extracellular gate (*39*). There is no similar intact pocket, akin to the allosteric 5-HT site in SERT, in the extracellular vestibule of LeuT or MhsT. Whether there is a second functionally relevant allosteric site in prokaryotic NSS homologs remains to be unambiguously resolved.

**Fig. 5.**
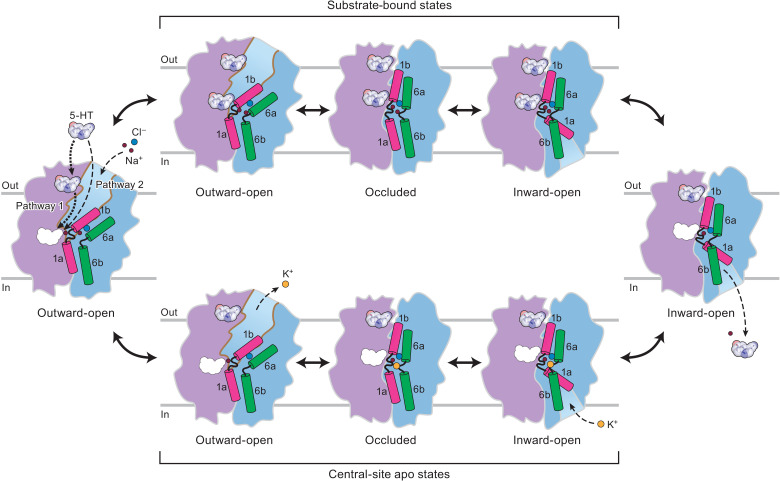
5-HT transport mechanism of SERT. Cartoon depicts the transport mechanism of SERT. The scaffold domain is in violet, and the core domain is in blue. TM1 and TM6 are highlighted in magenta and green, respectively. Na^+^, Cl^−^, and K^+^ are shown as red, cyan, and orange spheres, respectively.

By capturing 5-HT–bound SERT in different conformations, and by elucidating the conformational ensembles of SERT in apo states, in the presence of either Na^+^ or K^+^, we have determined the structural underpinnings of ion-coupled 5-HT transport (Fig. 5). We propose that transport is initiated when SERT is in an outward-facing conformation, with Na^+^ and Cl^−^ bound to their respective sites and 5-HT bound to the central site. The transporter then isomerizes to an inward-facing conformation via an occluded state, while the allosteric site, if occupied, retains bound 5-HT. This triggers unbinding of 5-HT from the central site, and a sodium ion, and their diffusion through the intracellular permeation pathway into the cytoplasm. After substrate release, the transporter cycles back to outward-facing via an occluded conformation, likely in an equilibrium between multiple states. When the transporter is in an outward-facing conformation, the central site can re-bind 5-HT, either from the extracellular solution or, perhaps more rapidly, from the allosteric 5-HT site, to prime the next round of transport (Fig. 5).

## MATERIALS AND METHODS

### Antibody production

The 15B8 Fab was produced by papain digestion of 15B8 monoclonal antibody and purified by cation exchange chromatography (*10*, *40*).

### MSP expression and purification

The pMSP1D1 plasmid encoding the polyhistidine-tagged membrane scaffold protein (MSP) was used to express the MSP, which was subsequently purified as described (*41*), with several modifications. Freshly transformed *Escherichia coli* cells (BL21 DE3) were grown in Terrific Broth (TB) medium containing kanamycin (50 mg liter^−1^) at 37°C with shaking (250 rpm). When the OD_600_ (optical density at 600 nm) reached ~2.5, the culture was induced with 1 mM isopropyl-β-d-thiogalactopyranoside (IPTG) and grown for 16 to 20 hours after induction at 20°C with shaking (250 rpm), after which it was harvested by centrifugation at 6238*g* for 15 min at 4°C.

The cell pellet was resuspended in 200 ml of 20 mM sodium phosphate buffer (pH 7.4) supplemented with 1 mM phenylmethylsulfonyl fluoride, 1% Triton X-100, and deoxyribonuclease I (30 mg liter^−1^) and lysed by sonication on ice. The lysate was centrifuged at 40,000 rpm for 30 min at 4°C (45 Ti fixed-angle rotor, Beckman). The supernatant was applied to a column packed with 15 ml of Ni–nitrilotriacetic acid (NTA) resin prepared in equilibration buffer [40 mM tris-HCl (pH 8), 300 mM NaCl, and 1% Triton X-100]. The resin was washed with 10 column volumes (CVs) of equilibration buffer, followed by 5 CVs of cholate buffer [40 mM tris-HCl (pH 8), 300 mM NaCl, 50 mM sodium cholate, and 20 mM imidazole] and 5 CVs of 40 mM tris-HCl (pH 8), 300 mM NaCl, and 50 mM imidazole. The MSP1D1 was eluted with 3 CVs of elution buffer [40 mM tris-HCl (pH 8), 300 mM NaCl, and 400 mM imidazole]. The eluted sample was dialyzed twice against 4 liters of a tris-buffered saline (TBS) buffer [20 mM tris-HCl (pH 8) and 100 mM NaCl] at 4°C to remove imidazole. The polyhistidine tag on MSP1D1 was removed by incubation with tobacco etch virus (TEV) protease in the presence of 10 mM dithiothreitol (DTT) overnight. The completeness of digestion was assessed by SDS–polyacrylamide gel electrophoresis. After complete digestion, the DTT and TEV protease were removed by passing the solution through a PD-10 column (Sigma-Aldrich) prepared in TBS buffer according to the manufacturer’s instructions. To remove the affinity tag, the sample was next passed through a gravity column with 5 ml of Ni-NTA resin, and the resin was washed with a buffer composed of 20 mM tris-HCl (pH 8), 100 mM NaCl, and 20 mM imidazole. The “flow-through” solution was collected, dialyzed against TBS to remove imidazole, and concentrated using a Millipore Ultra-15 filter (10-kDa cutoff). Final protein concentration was determined by ultraviolet absorbance at 280 nm. The concentrated MSP1D1 protein solution was aliquoted, flash-frozen in liquid nitrogen, and stored at −80°C until use.

### Preparation of phospholipids

A sample of brain total lipid (25 mg ml^−1^) was prepared by dissolving the powder in chloroform followed by evaporation and resuspension with 20 mM tris-HCl (pH 8) and 100 mM NaCl or 100 mM KCl. The solution was flushed with nitrogen and sonicated until clear (*41*).

### SERT expression, purification, and nanodisc reconstitution

The constructs used in this study are the N- and C-terminally truncated but otherwise wild-type (ΔN72, ΔC13) (*10*) and ts2-active (I291A, T439S) (*8*, *40*) proteins. The expression and purification of these constructs were carried out as described previously, with minor changes (*40*). The human SERT constructs were expressed as C-terminal green fluorescent protein (GFP) fusions using baculovirus-mediated transduction of mammalian human embryonic kidney (HEK)–293S GnTI^−^ cells (*42*). Cells were solubilized in 20 mM tris-HCl (pH 8) with 100 mM NaCl or 100 mM KCl, containing 20 mM *n*-dodecyl-β-d-maltoside (DDM) and 2.5 mM cholesteryl hemisuccinate (CHS), in the presence of 1 mM 5-HT, and then purified in 1 mM DDM, 0.2 mM CHS, and 1 mM 5-HT with either 100 mM NaCl or 100 mM KCl via affinity chromatography using Strep-Tactin resin.

For the ΔN72/C13 SERT–15B8 Fab complex with 5-HT, purified SERT was mixed with MSP1D1 and brain total lipid at a molar ratio of 1:5:500 and incubated on ice for 1 hour in the presence of 1 mM 5-HT. Thrombin was used to remove the N- and C-terminal GFP and purification tags. Next, the detergent was removed by incubation with Bio-Beads (500 to 600 mg ml^−1^) overnight at 4°C with gentle agitation. The 15B8 Fab was mixed with SERT at a 1:1.2 molar ratio, and the complex was purified by SEC on a Superdex 200 column in a buffer composed of 20 mM tris-HCl (pH 8) with 100 mM NaCl or 100 mM KCl and 1 mM 5-HT. The peak fraction containing the SERT complexes was concentrated to 5 to 6 mg ml^−1^ before the addition of 10 mM 5-HT.

For the apo ΔN72/C13 SERT–15B8 Fab complex, SERT was purified in a buffer composed of 1 mM DDM, 0.2 mM CHS, and 1 mM 5-HT with 100 mM NaCl and 20 mM tris-HCl (pH 8), mixed with MSP1D1 and brain total lipid at a molar ratio of 1:5:500, and allowed to incubate on ice for 1 hour. Thrombin was again used to remove the N- and C-terminal GFP and purification tags. Detergent was removed by incubation with Bio-Beads (500 to 600 mg ml^−1^) overnight at 4°C with gentle agitation. The 15B8 Fab was mixed with SERT at a 1:1.2 molar ratio and purified by SEC on a Superdex 200 column in a buffer composed of 20 mM tris-HCl (pH 8) with 100 mM NaCl or 100 mM KCl in the absence of 5-HT. The peak fraction containing the SERT complexes was concentrated to 5 to 6 mg ml^−1^.

### Cryo-EM sample preparation and data acquisition

The SERT-antibody complexes (2.5 μl), at a concentration of 50 to 60 μM, were applied to glow-discharged Quantifoil holey carbon grids (gold, 2.0/2.0 or 2.0/1.0 μm size/hole space, 200 mesh). For “multishot” data collection (*43*), 100 μM fluorinated *n*-octyl-β-d-maltoside (final concentration) was added to the sample before freezing. The grids were blotted for 1.0 to 2.0 s at 100% humidity at 20°C, followed by plunging into liquid ethane cooled by liquid nitrogen, using Vitrobot Mark IV. Dose-fractionated image stacks were recorded using a FEI Titan Krios operating at 300 keV, equipped with a Gatan energy filter. A Gatan K3 Summit direct electron detector was used to record movies in super-resolution counting mode on the Krios. The typical defocus values ranged from −1.0 to −2.5 μm. Each stack was exposed for 1 to 4.5 s and dose-fractionated into 61 or 80 frames, with a total dose of 50 to 60 e^−^ Å^−2^. Images were recorded using the automated acquisition programs SerialEM (*43*) or Leginon (*44*).

### Cryo-EM image processing

Micrographs were corrected for beam-induced drift using MotionCor2 (*45*). The defocus values were determined using Gctf (*46*). Particles were picked using DoG-Picker (*47*). Particles were subjected to reference-free 2D classification in cryoSPARC (*48*) followed by heterogeneous refinement. The 3D classification was performed in RELION-3.0 (*49*), with a soft mask excluding the constant domain of 15B8 Fab. Homogeneous refinement, local contrast transfer function (CTF) refinement, and nonuniform refinement were performed in cryoSPARC. The EM density map was sharpened using a negative B-factor that was automatically determined in cryoSPARC using a Guinier plot. The resolution of the reconstructions was assessed using the Fourier shell correlation (FSC) criterion and a threshold (*50*) of 0.143 in cryoSPARC. Local resolution of the final map was estimated in cryoSPARC.

For the ΔN72/C13 SERT–15B8 Fab complex with 5-HT in 100 mM NaCl, 10,116 movies were collected, yielding an initial set of 2,861,741 particles, which after two rounds of heterogeneous refinement left 1,038,367 particles (binned to a 100-pixel box, 2.592 Å pixel^−1^). After homogeneous refinement in cryoSPARC and 3D classification in RELION-3.0, 892,902 particles were reextracted (400-pixel box, 0.648 Å pixel^−1^) and subjected to local gctf refinement (per particle) (*46*), followed by homogeneous refinement, local CTF refinement (*48*), and nonuniform refinement in cryoSPARC. These particles were subjected to 3D classification in RELION-3.0 without image alignment (400-pixel box, 0.648 Å pixel^−1^) and were classified into two major classes. Particles with closed extracellular gate features and particles belonging to the class with open extracellular gate features were reextracted and subjected to homogeneous refinement, local CTF refinement, and nonuniform refinement in cryoSPARC, respectively (fig. S2A).

For the ΔN72/C13 SERT–15B8 Fab complex with 5-HT in 100 mM KCl, a total of 5,624,930 particles with a box size of 100 pixels (3.208 Å pixel^−1^) were selected from 16,033 micrographs. After one round of 2D classification and two rounds of heterogeneous refinement in cryoSPARC, 759,745 particles with clearly defined and recognizable features were combined for further 3D classification in RELION-3.0. Well-resolved classes containing 299,808 particles were reextracted (400-pixel box, 0.802 Å pixel^−1^) and further refined in cryoSPARC using homogeneous refinement, followed by local CTF refinement and nonuniform refinement (fig. S4A).

For the apo ΔN72/C13 SERT–15B8 Fab complex in 100 mM KCl, a total of 5,865,511 particles were selected from 19,294 movies. After one round of 2D classification and two rounds of heterogeneous refinement in cryoSPARC and 3D classification in RELION-3.0, particles belonging to a class with well-defined TM features were combined and reextracted (400-pixel box, 0.661 Å pixel^−1^). A total of 707,210 particles were subjected to homogeneous refinement, local CTF refinement, and nonuniform refinement (fig. S6A).

For the apo ΔN72/C13 SERT–15B8 Fab complex in 100 mM NaCl–containing buffer, two datasets were recorded with a calibrated pixel size of 0.648 Å pixel^−1^ (dataset I) and 0.636 Å pixel^−1^ (dataset II), respectively. To calculate the relative pixel size and scale the data, the pixel size of the dataset with the larger pixel size (dataset I) was kept constant while changing the relative pixel size of the second dataset (0.636 Å pixel^−1^, dataset II) (*51*). The micrographs from dataset II were rescaled to the final required pixel size of 0.648 Å pixel^−1^. A total of 2,299,295 particles were selected from 6794 movies of dataset I, and 3,269,136 particles were selected from 12,882 movies of dataset II, respectively. Following one round of 2D classification and two rounds of heterogeneous refinement in cryoSPARC, datasets I and II yielded 603,043 and 1,368,359 particles, respectively. After homogeneous refinement in cryoSPARC, these particles were combined and subjected to 3D classification in RELION-3.0 without image alignment (100-pixel box, 2.592 Å pixel^−1^). These particles were classified into two major classes. Particles with “closed” TM1a features were combined, and particles belonging to a class with “open” TM1a features were combined, which then were reextracted (400-pixel box, 0.648 Å pixel^−1^), respectively, and subjected to homogeneous refinement and local CTF refinement, followed by nonuniform refinement in cryoSPARC, yielding 3.9-Å map for both the closed and open conformations, respectively (fig. S7A).

### Model building and refinement

Interpretation of the cryo-EM maps exploited rigid-body fitting of the SERT-antibody complex models derived from previous cryo-EM studies (*10*). To build a structure for the ΔN72/C13 SERT–15B8 Fab complex with 5-HT in an outward-open conformation, the outward-open ibogaine ts2-active model (PDB code: 6ZDY) with removal of 8B6 variable domain served as a reference. For the ΔN72/C13 SERT–15B8 Fab complex in the inward-open conformation in NaCl, in KCl, or in the presence of 5-HT, the ibogaine-bound SERT structure in an inward-open conformation (PDB code: 6DZZ) was used as an initial model. For the molecular model of the ΔN72/C13 SERT–15B8 Fab complex in an occluded conformation in NaCl, the occluded conformation of SERT (PDB code: 6DZV) was docked into the electron density using UCSF Chimera (*52*). The model building involved manual adjustments and building where merited by the quality of the EM maps in Coot (*53*), followed by real-space refinement in PHENIX (*54*). MolProbity was used to evaluate the stereochemistry and geometry of the structures (*55*). For all the ΔN72/C13 SERT–15B8 Fab complexes, SERT residues 79 to 617 were modeled into the cryo-EM maps, except for the apo ΔN72/C13 SERT–15B8 Fab complex in the occluded conformation, where SERT residues 79 to 615 were fit into the cryo-EM map. Figures were prepared in PyMOL (*56*) and UCSF Chimera (*57*).

### Radioligand binding and uptake assays

All the competition-binding experiments were performed using scintillation proximity assays (SPAs) (*58*) with Cu-YSi beads (1 mg ml^−1^) in SPA buffer containing 20 mM tris-HCl (pH 8), 100 mM NaCl, or 100 mM KCl. For the [^3^H]ibogaine assays, SPA was performed with 50 nM SERT, 300 nM [^3^H]ibogaine, and 1 μM to 10 mM cold 5-HT. For the [^3^H]paroxetine experiments, 1 nM SERT was used along with 0.3 μM to 10 mM of the cold competitors and 20 nM [^3^H]paroxetine. For the [^3^H]VLZ binding experiments, SPA was done with 20 nM SERT and 800 nM [^3^H]VLZ in the presence of 800 nM imipramine at 1 μM to 3 mM cold competitors. Experiments were measured in triplicate. The error bars for each data point represent the SEM *K*i values and were determined with the Cheng-Prusoff equation (*59*) in GraphPad Prism.

Ibogaine binding was measured via SPA using the ΔN72/C13 SERT protein purified in SPA buffer. Each experiment contained SERT at a concentration of 50 nM mixed with Cu-YSi beads (1 mg ml^−1^) and [^3^H]ibogaine at a concentration of 62.5 to 2000 nM (1:10 hot:cold). The [^3^H]paroxetine-binding experiments were performed with 1 nM SERT mixed with Cu-YSi beads (1 mg ml^−1^) in SPA buffer, and [^3^H]paroxetine was at a concentration of 0.3 to 40 nM. Nonspecific binding was estimated by experiments that included 100 μM unlabeled paroxetine or *S*-citalopram. Data were analyzed using a single-site binding function.

Vilazodone binding was measured in the presence of cold imipramine via SPA using ts2-active SERT purified in SPA buffer. Each experiment contained SERT at a concentration of 20 nM mixed with Cu-YSi beads (1 mg ml^−1^), [^3^H]VLZ at a concentration of 31.3 to 4000 nM in SPA buffer, and cold imipramine at a concentration of 31.3 to 4000 nM. Nonspecific binding was estimated by experiments that included 100 μM unlabeled *S*-citalopram. Data were analyzed using a single-site binding function.

To measure the uptake of the *N*-indole methyl derivative, 1 × 10^5^ HEK-293S GnTI^−^ cells were transduced with viruses for ΔN72/C13 SERT. The cells were plated into 96-well plates coated with poly-d-lysine. After 24 hours, cells were washed with uptake buffer [25 mM Hepes-tris (pH 7), 130 mM NaCl, 5.4 mM KCl, 1.2 mM CaCl_2_, 1.2 mM MgSO_4_, 1 mM ascorbic acid, and 5 mM glucose]. The custom-synthesized [^3^H]3-(2-aminoethyl)-1-methyl-1*H*-indol-5-ol hydrochloride isotope was diluted 1:1000 with unlabeled ligand, and a final concentration 0.78 to 100 μM was added to the cells. Uptake was stopped at 5 min by rapidly washing cells three times with 100 μl of uptake buffer, solubilizing with 20 μl of 1% Triton-X100, followed by addition of 200 μl of scintillation fluid to each well. A final concentration of 100 μM *S*-citalopram was added to the cells to estimate nonspecific counts. The amount of labeled 3-(2-aminoethyl)-1-methyl-1*H*-indol-5-ol hydrochloride was measured by counting in a standard 96-well plate using a MicroBeta scintillation counter.
