# Supplementary material for: Illumination of serotonin transporter mechanism and role of the allosteric site
Source: Sci Adv. 2021 Dec 1;7(49):eabl3857. doi: 10.1126/sciadv.abl3857 (PMC8635421; doi:10.1126/sciadv.abl3857)
Supplement: Supplementary file 1 — Figs. S1 to S7 [file sciadv.abl3857_sm.pdf]

Science Advances
AAAS

# Supplementary Materials for

## Illumination of serotonin transporter mechanism and role of the allosteric site

Dongxue Yang and Eric Gouaux*

*Corresponding author. Email: gouauxe@ohsu.edu

Published 1 December 2021, *Sci. Adv.* **7**, eabl3857 (2021)
DOI: 10.1126/sciadv.abl3857

**This PDF file includes:**

Figs. S1 to S7

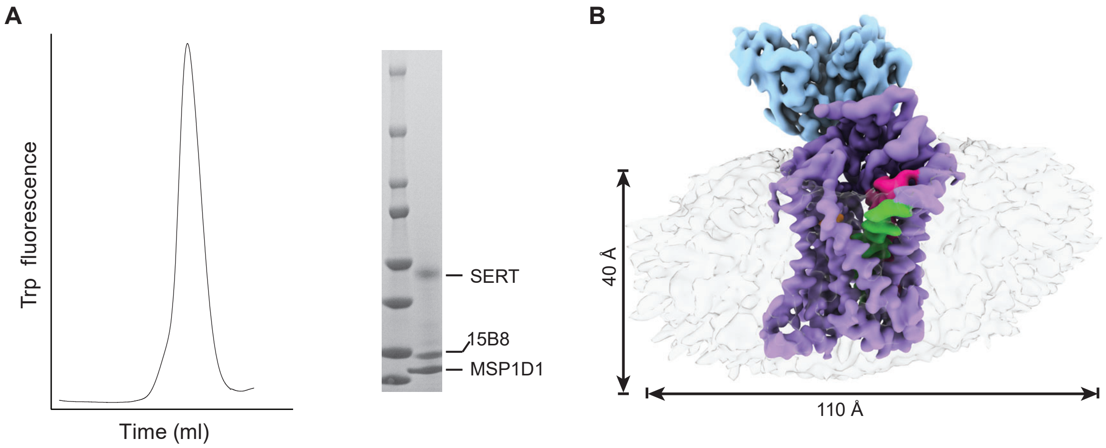


**Fig. S1. Reconstitution of SERT into nanodiscs. (A)** The SEC profile of the SERT-15B8-Fab complex in the presence of 5-HT in MSP1D1 nanodiscs and the SDS-PAGE gel of the purified complex protein after SEC. The gel was repeated three times from different batches of purification and similar results were obtained. (**B**) The initial reconstruction of the SERT-5HT complex in nanodiscs in NaCl before 3D classification. The density associated with the nanodisc is shown as a partially transparent surface.

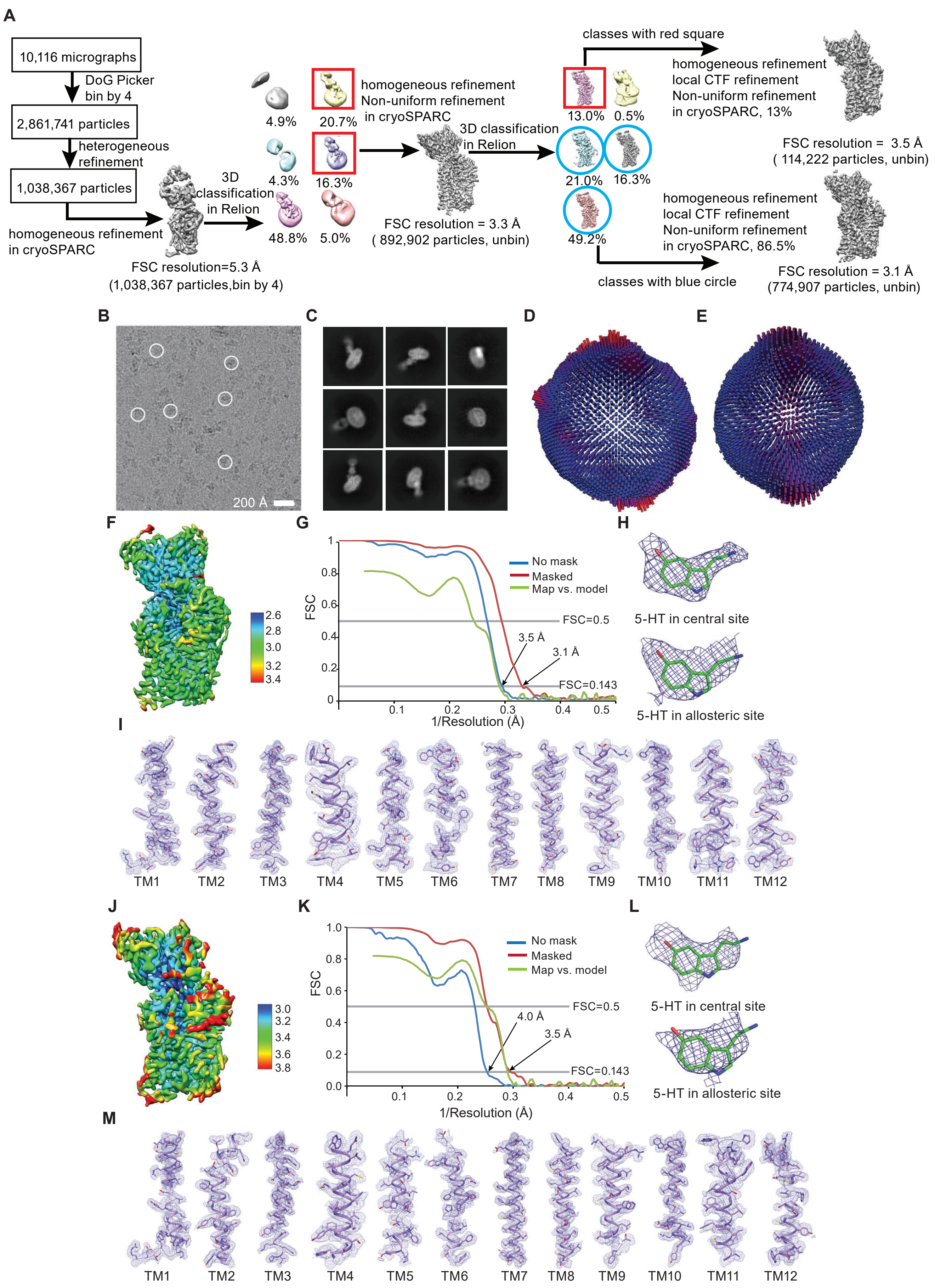
A
10,116 micrographs
DoG Picker
bin by 4
2,861,741 particles
heterogeneous
refinement
1,038,367 particles
homogeneous refinement
in cryoSPARC
FSC resolution=5.3 Å
(1,038,367 particles,bin by 4)
3D
classification
in Relion
4.9%
20.7%
4.3%
16.3%
48.8%
5.0%
homogeneous refinement
Non-uniform refinement
in cryoSPARC
FSC resolution = 3.3 Å
( 892,902 particles, unbin)
3D classification
in Relion
classes with red square
13.0%
0.5%
21.0%
16.3%
49.2%
homogeneous refinement
local CTF refinement
Non-uniform refinement
in cryoSPARC, 13%
FSC resolution = 3.5 Å
( 114,222 particles, unbin)
homogeneous refinement
local CTF refinement
Non-uniform refinement
in cryoSPARC, 86.5%
FSC resolution = 3.1 Å
(774,907 particles, unbin)
classes with blue circle
B
200 Å
C
D
E
F
2.6
2.8
3.0
3.2
3.4
G
FSC
1/Resolution (Å)
No mask
Masked
Map vs. model
FSC=0.5
FSC=0.143
3.5 Å
3.1 Å
H
5-HT in central site
5-HT in allosteric site
I
TM1
TM2
TM3
TM4
TM5
TM6
TM7
TM8
TM9
TM10
TM11
TM12
J
3.0
3.2
3.4
3.6
3.8
K
FSC
1/Resolution (Å)
No mask
Masked
Map vs. model
FSC=0.5
FSC=0.143
4.0 Å
3.5 Å
L
5-HT in central site
5-HT in allosteric site
M
TM1
TM2
TM3
TM4
TM5
TM6
TM7
TM8
TM9
TM10
TM11
TM12

**Fig. S2. Cryo-EM reconstructions of the 5-HT-SERT complex in NaCl.** (**A**) Flow chart of cryo-EM data analysis for the 5-HT-SERT complex in NaCl. (**B**) A representative micrograph, with single particles circled (white). (**C**) 2D class averages. (**D** and **E**) Angular distribution of particles used for the final 3D reconstructions of the outward (**D**) and occluded (**E**) conformations. (**F** to **I**) Cryo-EM density map colored by local resolution estimation (**F**), map-map and map-model Fourier shell correlation (FSC) curves (**G**), cryo-EM densities for 5-HT in the central and allosteric sites (**H**), cryo-EM density corresponding to TM1-TM12 (**I**) for the outward conformation. (**J** to **M**) Local-resolution distribution of the final map (**J**), map-map and map-model FSC curves (**K**), 5-HT densities (**L**) and the densities corresponding to TM1-TM12 (**M**) for the occluded conformation.

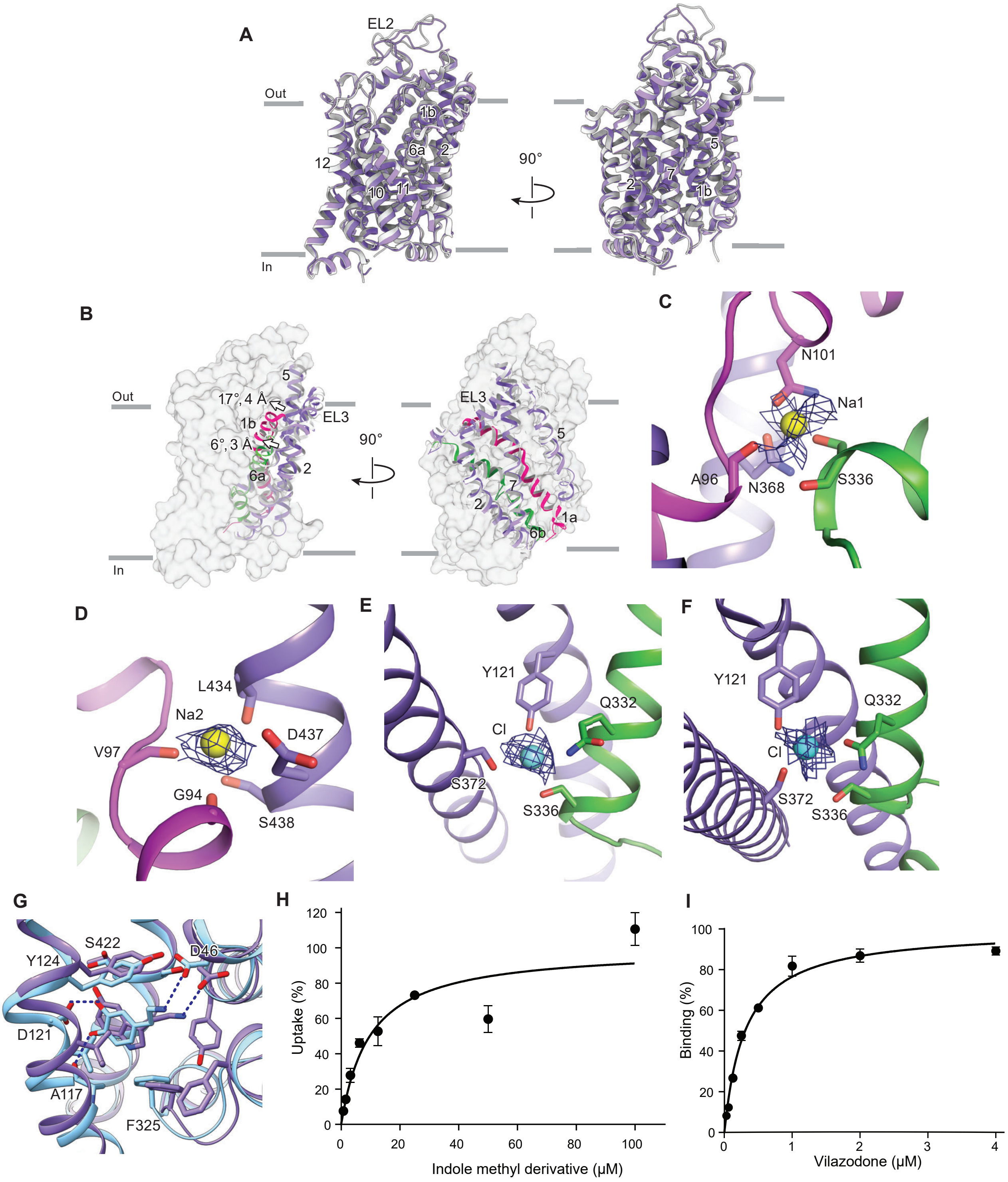
A
EL2
Out
In
1b
6a
2
12
10
11
90°
5
7
2
1b
B
Out
In
5
17°, 4 Å
1b
6°, 3 Å
EL3
6a
2
90°
EL3
5
7
2
1a
6b
C
N101
Na1
A96
N368
S336
D
L434
Na2
D437
V97
G94
S438
E
Y121
Q332
Cl
S372
S336
F
Y121
Q332
Cl
S372
S336
G
S422
D46
Y124
D121
A117
F325
H
Uptake (%)
Indole methyl derivative (μM)
0
20
40
60
80
100
120
I
Binding (%)
Vilazodone (μM)
0
1
2
3
4
20
40
60
80
100

**Fig. S3. Comparison of the 5-HT-SERT complex in NaCl to the S-citalopram-bound outward and ibogaine-bound occluded conformations, and the ion densities in the 5-HT-SERT complex in NaCl. (A**) Superposition of the 5-HT-bound outward (purple) with the S-citalopram bound outward (PDB code: 5I73, grey) conformations. (**B**) Superposition of the 5-HT-SERT occluded and ibogaine-SERT occluded (PDB: 6DZV, grey) conformations. The scaffold domain is shown in surface representation and movements of TM1b (magenta) and TM6a (green) are indicated. (**C** and **D**) The sodium densities found in the 5-HT-SERT complex in the outward conformation. (**E** and **F**) The chloride densities found in the outward (**E**) and occluded (**F**) conformations. (**G**) Superposition of central binding pocket of dopamine-DAT structure (PDB code: 4XP1, cyan) with central binding site of 5-HT–SERT (purple). Residues interacting with dopamine have been indicated. Hydrogen bond interactions are represented as dashed lines. (**H**) Uptake experiments using [$^{3}$H]-3-(2-aminoethyl)-1-methyl-1H-indol-5-ol hydrochloride yielded a Michaelis constant ($K_m$) of 9.7± 4.4 µM. Symbols show the mean derived from n=3 biological replicates. Error bars show the s.e.m. The experiment was performed twice independently with similar results. (**I**) [$^{3}$H]VLZ saturation binding experiments of SERT in nanodiscs in NaCl and corresponding mean $K_d$ values derived from the curve fit. Symbols show the mean derived from n=3 biological replicates. Error bars show the s.e.m.

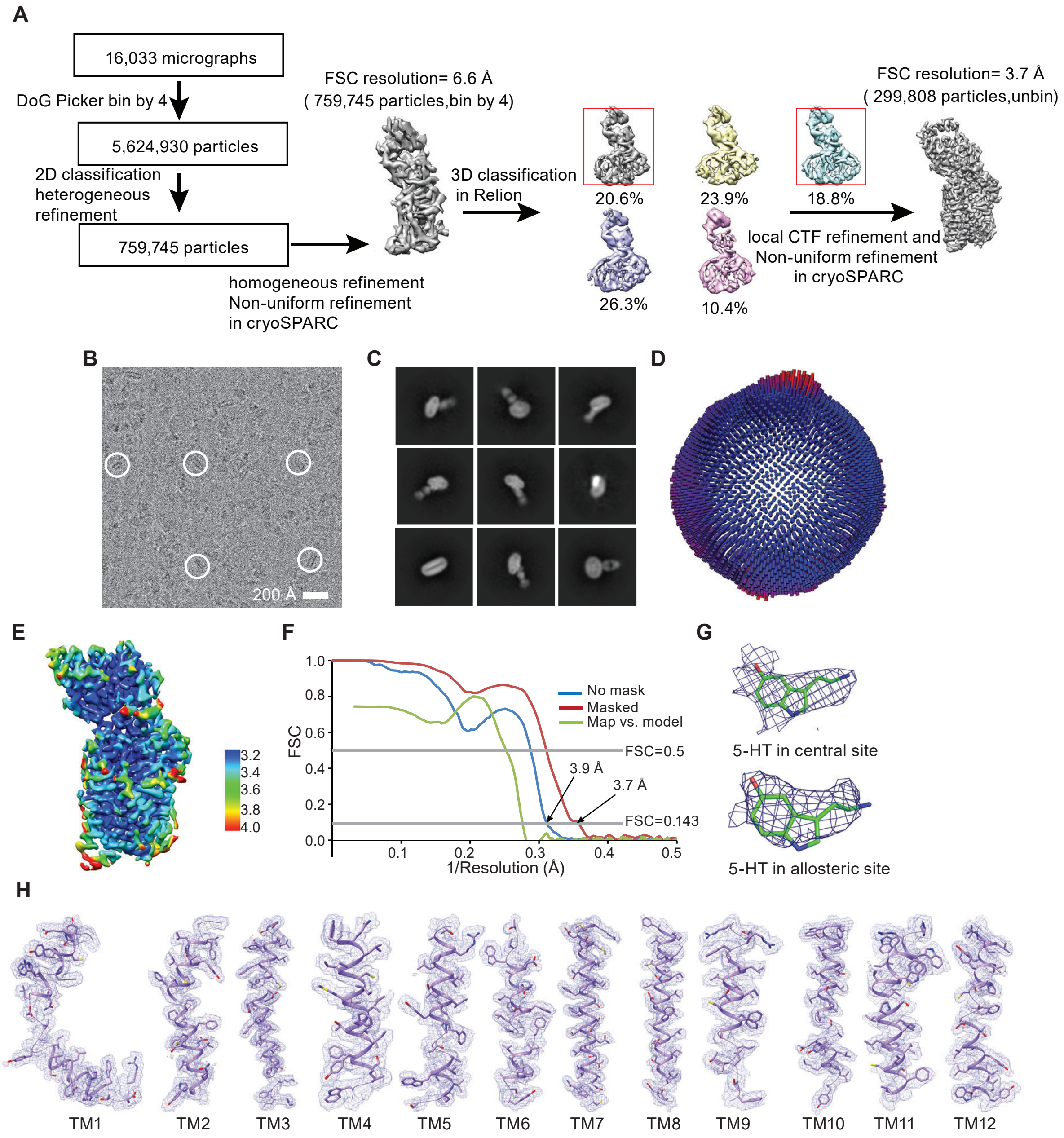


**Fig. S4. Structure determination of the 5-HT-SERT complex in KCl.** (**A**) Workflow for the determination of the structure of the 5-HT-SERT complex in KCl. (**B)** Representative micrograph, with individual single particles identified by white circles. (**C**) 2D class averages. (**D**) Angular distribution of particles used for the final 3D reconstruction. (**E**) Local resolution distribution of the final map. (**F)** Map-map and map-model FSC curves. (**G**) Cryo-EM densities for 5-HT. (**H**) Cryo-EM density segments for TM1-TM12.

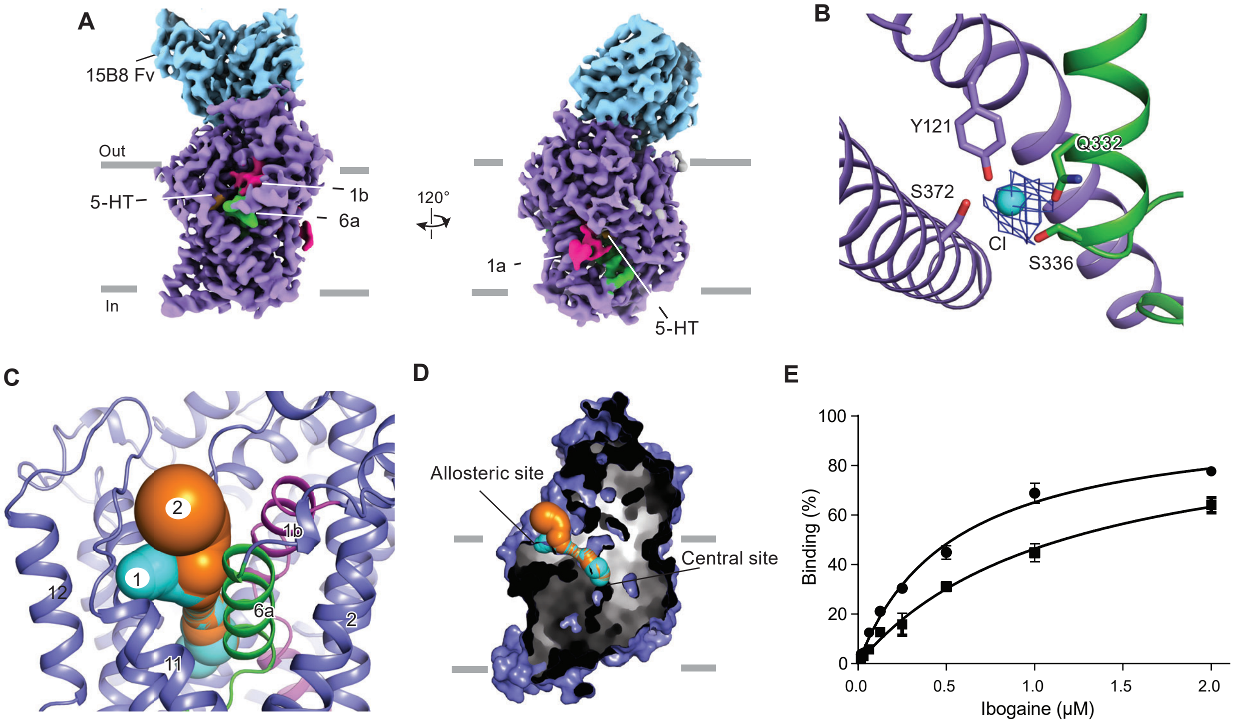


**Fig. S5. The cryo-EM density map of 5-HT-bound SERT in KCl and the chloride density, pathways to the central binding site in the outward-facing conformation, and ibogaine binding with apo SERT in nanodiscs in NaCl and KCl. (A)** The cryo-EM density map of the 5-HT-SERT complex in KCl. (**B**) The chloride density found in the 5-HT-SERT complex in KCl. (**C**) Solvent-accessible pathways in the outward-facing conformation. Pathway 1 leads from the allosteric site to the central site. Pathway 2 leads from the extracellular solution to the central site. (**D**) The slab view of the 'tunnels' from the central site to the allosteric site and extracellular space. (**E**) [$^3$H]-Ibogaine saturation binding experiments of SERT in nanodiscs in NaCl (squares, $K_d$ = 1160± 150 nM) and in KCl (circles, $K_d$ = 535± 68 nM). Symbols show the mean derived from n=3 biological replicates. Error bars show the s.e.m.

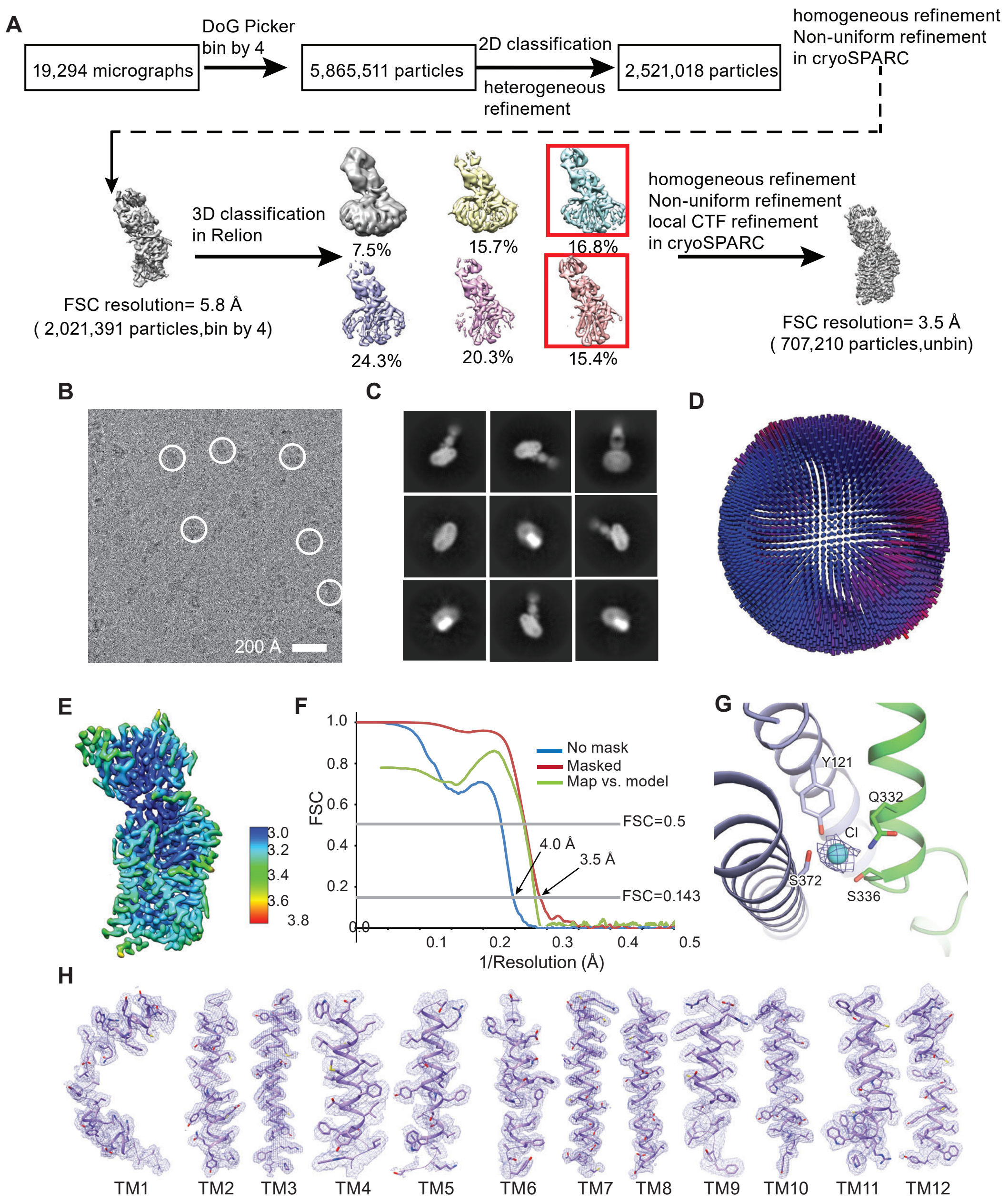


**Fig. S6. The cryo-EM structure of apo SERT in KCl. (A)** Workflow for the determination of the structure of apo SERT in KCl. (**B**) Representative micrograph with individual single particles circled in white. (**C**) 2D class averages. (**D**) Angular distribution of particles used for the final 3D reconstruction. (**E**) Local resolution for the density map. (**F**) Map-map and map-model FSC curves. (**G**) Chloride density. (**H**) Cryo-EM density segments of TM1-TM12.

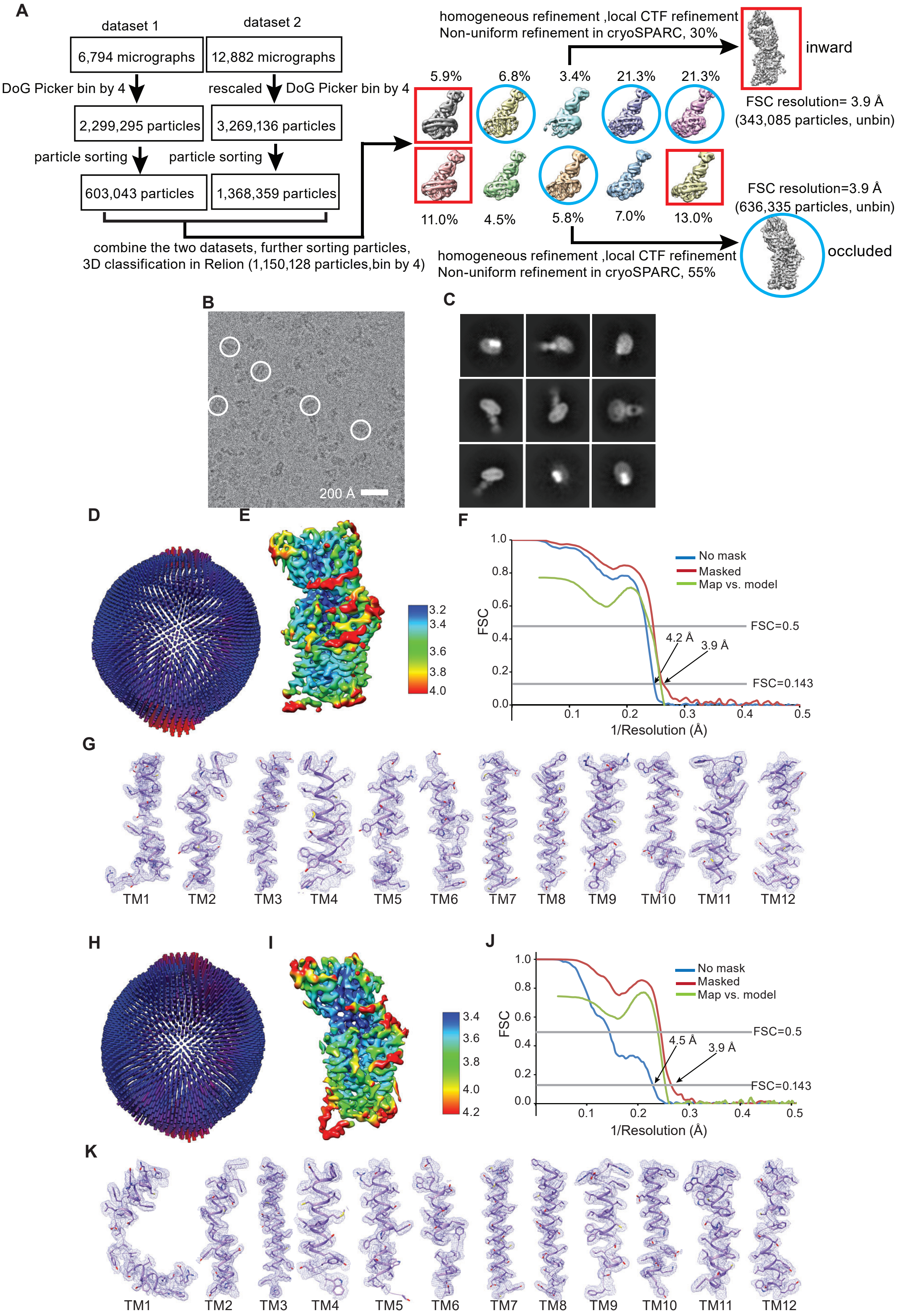
A
dataset 1
dataset 2
6,794 micrographs
12,882 micrographs
DoG Picker bin by 4
rescaled
DoG Picker bin by 4
2,299,295 particles
3,269,136 particles
particle sorting
particle sorting
603,043 particles
1,368,359 particles
combine the two datasets, further sorting particles,
3D classification in Relion (1,150,128 particles,bin by 4)
homogeneous refinement ,local CTF refinement
Non-uniform refinement in cryoSPARC, 30%
inward
FSC resolution= 3.9 Å
(343,085 particles, unbin)
5.9%
6.8%
3.4%
21.3%
21.3%
11.0%
4.5%
5.8%
7.0%
13.0%
FSC resolution=3.9 Å
(636,335 particles, unbin)
homogeneous refinement ,local CTF refinement
Non-uniform refinement in cryoSPARC, 55%
occluded
B
200 Å
C
D
E
3.2
3.4
3.6
3.8
4.0
F
No mask
Masked
Map vs. model
FSC
FSC=0.5
FSC=0.143
4.2 Å
3.9 Å
1/Resolution (Å)
G
TM1
TM2
TM3
TM4
TM5
TM6
TM7
TM8
TM9
TM10
TM11
TM12
H
I
3.4
3.6
3.8
4.0
4.2
J
No mask
Masked
Map vs. model
FSC
FSC=0.5
FSC=0.143
4.5 Å
3.9 Å
1/Resolution (Å)
K
TM1
TM2
TM3
TM4
TM5
TM6
TM7
TM8
TM9
TM10
TM11
TM12

**Fig. S7. Structure determination of apo SERT in NaCl.** (**A**) Workflow for the determination of the structure of apo SERT in NaCl. (**B**) Representative micrograph with individual single particles circled in white. (**C**) 2D class averages. (**D** to **G**) Angular distribution of particles used for the final 3D reconstruction (**D**), cryo-EM density map colored by local resolution estimation (**E**), map-map and map-model FSC curves (**F**), cryo-EM density segments of TM1-TM12 (**G**), for the occluded conformation. (**H** to **K**) Angular distribution of particles used for the final 3D reconstruction (**H**), local-resolution distribution of the final map (**I**), map-map and map-model FSC curves (**J**), density fitting of TM1-TM12 (**K**), for the inward conformation.
